# Predicting Long-Term Mortality Using American College of Cardiology (ACC)/American Heart Association (AHA) Pooled Cardiovascular Risk Cohort Equations: Implications for Lipid-Lowering Therapy

**DOI:** 10.7759/cureus.72856

**Published:** 2024-11-01

**Authors:** Zhiyuan Ma, Lynn N Moran, Jamshid Shirani

**Affiliations:** 1 Department of Cardiology, St Luke's University Health Network, Bethlehem, USA

**Keywords:** all-cause mortality, ascvd risk, cardiovascular mortality, lipid-lowering therapy, risk assessment

## Abstract

Introduction: Primary prevention statin therapy is initiated based on the 10-year ASCVD risk estimated by the pooled cohort equations (PCEs), with treatment effectiveness monitored through low-density lipoprotein cholesterol levels. It remains uncertain whether ASCVD risk accurately predicts the mortality impact of lipid-lowering therapy. This study aims to assess the predictive performance of the PCE for mortality with and without lipid-lowering therapy and the mortality impact of lipid-lowering therapy with similar ASCVD risk.

Methods: A retrospective analysis of the National Health and Nutrition Examination Survey (NHANES) (III and 1999-2008) linked to the National Death Index was conducted with propensity score matching. Cox regression and C statistic were used to determine the association between 10-year ASCVD risk and mortality.

Results: In the matched cohort with 4,802 individuals with similar ASCVD risks, the 10-year risk of ASCVD by PCE was comparable for predicting all-cause mortality at 10 years in the lipid-lowering therapy group (area under the curve (AUC), 0.75; 95% confidence interval (CI) 0.73-0.77) and without lipid-lowering therapy (0.74; 95% CI 0.71-0.77; p = 0.50). Similarly, PCE was comparable for predicting cardiovascular mortality at 10 years in both groups (AUC, 0.75; 95% CI 0.70-0.79 versus 0.77; 95% CI 0.73-0.80; p = 0.47). Lipid-lowering therapy was significantly associated with reduced all-cause mortality (adjusted hazard ratio (HR), 0.70; 95% CI 0.61-0.82; p < 0.01) and cardiovascular mortality (adjusted HR, 0.65; 95% CI, 0.51-0.83; p < 0.01), particularly in those with a 10-year ASCVD risk of 7.5% or higher.

Conclusion: The PCE comparably predicts mortality for both those on and off lipid-lowering therapy. Those on therapy have lower all-cause and cardiovascular mortality despite similar ASCVD risk. This underscores the usefulness of PCE in assessing mortality risk, both before and after treatment.

## Introduction

Atherosclerotic cardiovascular disease (ASCVD) is a leading cause of morbidity and mortality worldwide [1,2] and affects about 26 million people in the United States [3,4]. The primary strategy for preventing ASCVD involves reducing the risk of developing ASCVD through lifestyle changes and the use of lipid-lowering therapy, such as statin therapy [5]. Current guidelines recommend the use of the 10-year risk of ASCVD calculated from the pooled cohort equations (PCEs) [6] to identify individuals at high risk of ASCVD who would benefit most from statin therapy [7-10]. Statin therapy is recommended for adults aged 40 to 75 years with a 10-year risk of ASCVD of 7.5% or higher, with diabetes, or with low-density lipoprotein cholesterol (LDL-C) ≥ 190 mg/dL, and for those with clinical ASCVD, encompassing conditions like acute coronary syndrome, a history of myocardial infarction, stable or unstable angina, coronary or other arterial revascularization, stroke, transient ischemic attack, or peripheral artery disease, all stemming from atherosclerotic origins [6,8]. Many clinical trials have demonstrated that statin therapy reduces the risk of ASCVD and improves all-cause and cardiovascular mortality in individuals with varying baseline risks [11-14], particularly those with elevated LDL-C levels [15]. In clinical practice, the effectiveness of statin therapy is typically assessed through the monitoring of LDL-C levels. In general, reducing LDL-C levels by 1.0 mmol/L (about 39 mg/dL) is associated with about 25% reduction in the risk of cardiovascular disease (CVD) events [16,17].

While the PCE is widely used to estimate 10-year ASCVD risk and guide statin therapy initiation, the ability of the 10-year ASCVD risk derived from the PCE to predict mortality outcomes in individuals receiving lipid-lowering therapy and monitor the effectiveness of statin therapy has not been explored. Specifically, it is unclear whether the PCE can equally predict mortality in individuals on lipid-lowering therapy compared to those not on therapy and whether the PCE can be used to assess the mortality benefits of lipid-lowering therapy in individuals with similar ASCVD risk profiles. In this study, we aimed to evaluate the performance of the PCE in predicting all-cause and cardiovascular mortality in individuals both receiving and not receiving lipid-lowering therapy and to determine the impact of lipid-lowering therapy on mortality in those with similar ASCVD risk using data from the National Health and Nutrition Examination Survey (NHANES) (III and 1999-2008) linked to the National Death Index.

This article was previously posted to the medRxiv preprint server on April 9, 2024.

## Materials and methods

Study population

For this analysis, data from NHANES III and continuous NHANES 1999-2008 were included, in which civilian noninstitutionalized individuals in the United States were surveyed (https://www.cdc.gov/nchs/nhanes/index.htm). All individuals provided informed consent to participate in the NHANES study. The included NHANES study was approved by the NHANES Institutional Review Board and the NCHS Research Ethics Review Board. All survey data are deidentified and publicly available. This study was considered exempt from local institutional research board review. This study followed the Strengthening the Reporting of Observational Studies in Epidemiology (STROBE) reporting guideline [18]. Adults aged 40 to 79 were included in the study. We excluded individuals with a history of myocardial infarction, heart failure, coronary artery disease, or stroke; those with missing data for high-density lipoprotein cholesterol, total cholesterol (TC), systolic blood pressure (SBP), or body mass index (BMI) or ineligible for mortality linkage; and those for whom 10-year ASCVD risk calculation was not feasible due to SBP < 90 mmHg or >200 mmHg or TC < 130 mg/dL or >320 mg/dL. Individuals in the NHANES survey with missing self-reported responses regarding antihypertensive or lipid-lowering medications were classified as not receiving therapy.

Variable definitions

Hypertension was defined as SBP ≥ 140 mmHg or diastolic blood pressure ≥ 90 mmHg or self-reported use of antihypertensive medication. Diabetes was defined as hemoglobin A1c concentration ≥ 6.5% or a self-reported diagnosis of diabetes. We calculated the 10-year risk of ASCVD based on the 2018 American College of Cardiology (ACC)/American Heart Association (AHA) guidelines [8] by the PCE [6].

Outcome measures

The primary outcome was all-cause mortality, with follow-up limited to 10 years. Every participant was followed for at least 10 years unless they experienced mortality within that period. The secondary outcome was cardiovascular mortality. Mortality follow-up data were obtained from the Linked Mortality Files, which were collected from death certificate records from the National Death Index through December 31, 2019, using a probabilistic linkage algorithm. Underlying Cause of Death Recodes (UCOD_113) in the Linked Mortality Files was created to assist mortality analyses using the International Classification of Diseases, 9th Revision (ICD-9) and 10th Revision (ICD-10) codes. Death from cardiovascular diseases, which were derived from UCOD_113 codes (054-068 and 070), consisted of deaths from diseases of the heart (I00-I09, I11, I13, and I20-I51), including ischemic heart disease (I20 through I25), heart failure (I50) and essential hypertensive heart disease (I11 through I13), and cerebrovascular disease (I60 through I69).

Statistical analysis

The discriminatory power of the risk of mortality as a function of 10-year ASCVD risk with or without lipid-lowering therapy was assessed by the area under the receiver-operating characteristic curve (AUC) (C statistic). The AUCs of the models with or without lipid-lowering therapy were compared for statistical differences with the use of bootstrapping.

To account for potential confounders and ensure the robustness of our results, we performed propensity score matching based on variables such as age, BMI, gender, race, education, and 10-year ASCVD risk. Propensity scores were estimated using a multivariable logistic regression model. We created a matched cohort using a 1:1 ratio with a caliper size of 0.05 to ensure close matching. The balance between covariates was assessed by estimating standardized mean differences (SMDs) for each variable to confirm that they were well matched. SMD < 0.1 is considered a negligible group imbalance [19]. Sensitivity analyses were conducted using the unmatched cohort.

The Kaplan-Meier method was used to estimate the cumulative event rate, and competing risks were modeled for cardiovascular mortality and death from other causes for the secondary outcome [20]. Individuals were categorized based on their 10-year ASCVD risk according to the 2018 ACC/AHA guidelines (<5%, ≥5% to 7.4%, ≥7.5% to 19.9%, and ≥20%) for subgroup analysis. The relative risk of mortality across different ASCVD risk groups, with or without lipid-lowering therapy, was assessed using Cox proportional hazards models, with the <5% ASCVD risk group serving as the reference. Models were adjusted for potential confounders including age, race, hypertension, diabetes, smoking, and 10-year ASCVD risk to evaluate the association between lipid-lowering therapy and mortality outcomes. The unadjusted Cox regression was used to compare the treatment effect between each category, and the interaction between lipid-lowering therapy status and 10-year ASCVD risk was tested using interaction terms in the unadjusted Cox regression.

Data were presented as the mean and 95% confidence interval (CI) or median and interquartile range (IQR) for continuous variables. For differences in continuous variables, Student’s t tests were used in two groups for comparison. Categorical variables were expressed as percentages (%), and weights for examination and interview portions of the survey were applied to account for the complex multistage probability-sampling design, survey nonresponse, and oversampling. For the comparison of categorical variables, χ^2^ tests were used. The risk of outcome was calculated as hazard ratios (HRs) and 95% CI. Statistical significance was determined at p-values <0.05. All analyses were conducted with R software, version 4.1.2 (R Foundation for Statistical Computing, Vienna, Austria).

## Results

**Figure 1:**
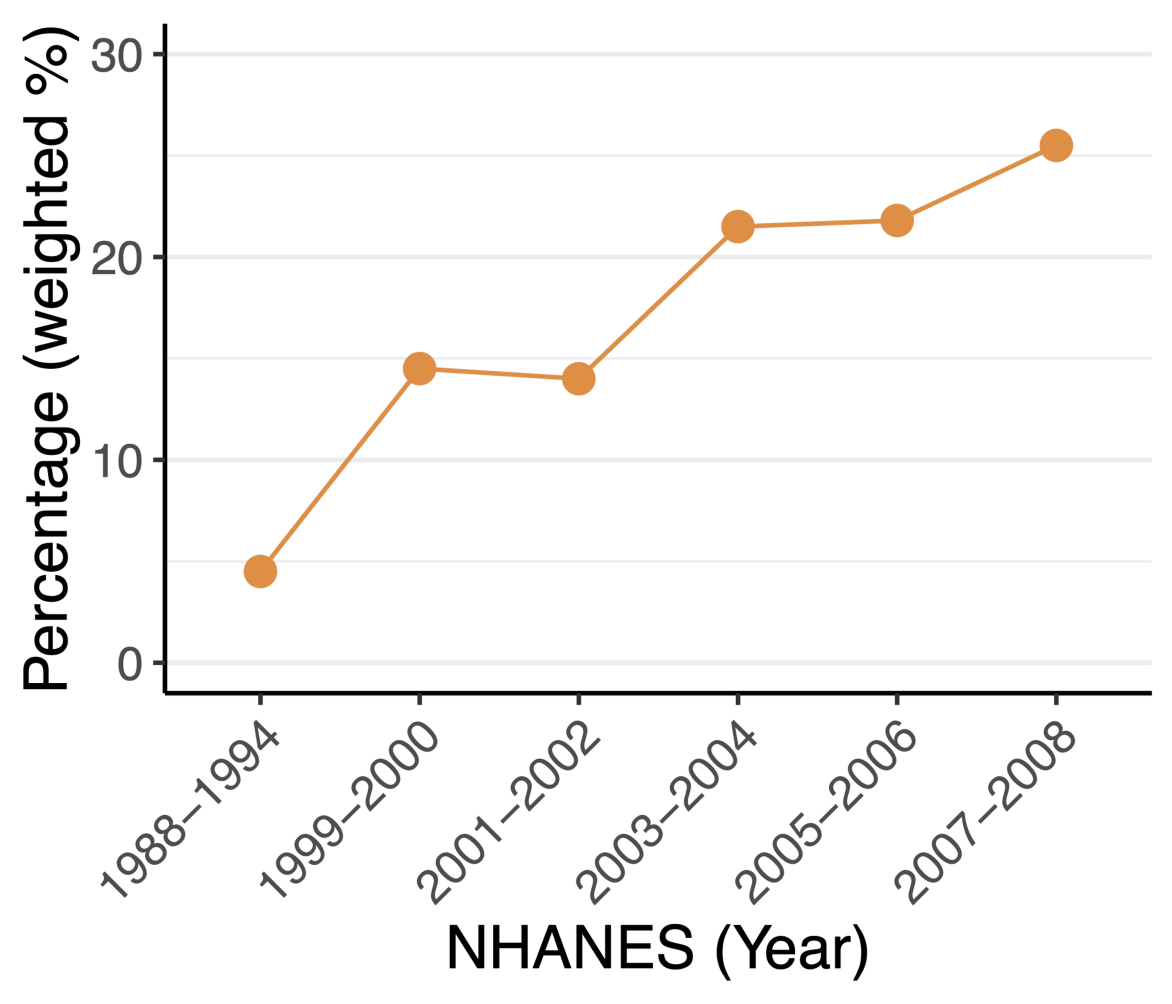
The weighted percentage of lipid-lowering therapy in the NHANES from 1988 to 2008 NHANES: National Health and Nutrition Examination Survey

**Figure 2:**
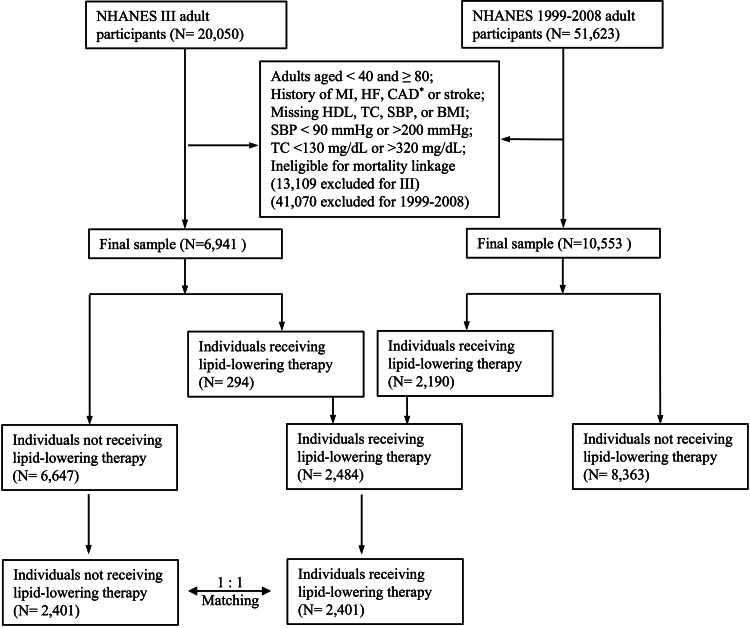
Flowchart illustration of the study cohorts BMI: body mass index; CAD: coronary artery disease; HDL: high-density lipoprotein cholesterol; HF: heart failure; MI: myocardial infarction; NHANES: National Health and Nutrition Examination Survey; SBP: systolic blood pressure; TC: total cholesterol ^*^CAD was not specified in NHANES III

A total of 6,647 individuals without lipid-lowering therapy (47.4% men, median age 56 years) from NHANES III and 2,484 with lipid-lowering therapy (47.1% men, median age 62 years) were matched based on similar 10-year risk of ASCVD, resulting in a cohort of 4,802 individuals. Individuals receiving lipid-lowering therapy had higher rates of hypertension (64.3% versus 47.3%, p < 0.01) and diabetes (27.0% versus 16.5%, p < 0.01) and lower rates of smoking (14.5% versus 18.0%, p < 0.01), compared with those not receiving lipid-lowering therapy. The lipid-lowering therapy group had a lower 10-year all-cause mortality rate (13.4%, 321 of 2,401) and cardiovascular mortality rate (4.4%, 106 of 2,401), compared with those without lipid-lowering therapy (19.0%, 456 of 2,401 and 6.9%, 165 of 2,401, respectively). Moreover, a significant increase in lipid-lowering therapy was noted in the USA from the period 1984-1994 to the early 2000s (Figure 1). The flow diagram of patient selections is shown in Figure 2. The baseline characteristics of cohorts from NHANES III and 1999-2008 are summarized in Table 1.

**Table 1:** Baseline characteristics of individuals by lipid-lowering therapy status ASCVD: atherosclerotic cardiovascular disease; BMI: body mass index; IQR: interquartile range ^a^Percentage is derived from the cohort after match; ^b^excluding the category of "unknown"

	NHANES III		NHANES 1999-2008		After match^a^		
Characteristic	Untreated (control)	Lipid-lowering therapy	Untreated (control)	Lipid-lowering therapy	Untreated (control)	Lipid-lowering therapy	p-value
No. in sample	6,647	294	8,363	2,190	2,401	2,401	
Age, median (IQR)	56 (46-66)	64 (57-70)	54 (46-64)	62 (53-70)	63 (53-71)	62 (53-69)	0.074
Male, no. (weighted %)	3,150 (46.6)	122 (44.9)	4,111 (46.7)	1,048 (49.0)	1,105 (46.0)	1,130 (47.1)	0.487
Race, no. (weighted %)							0.932
Mexican American	1,630 (3.6)	51 (2.0)	1,847 (5.8)	405 (4.3)	439 (18.3)	453 (18.9)	
Non-Hispanic Black	1,677 (9.0)	57 (5.8)	1,677 (9.8)	377 (7.5)	427 (17.8)	430 (17.9)	
Non-Hispanic White	3,070 (80.8)	177 (85.1)	4,082 (75.7)	1,203 (79.1)	1,359 (56.6)	1,338 (55.7)	
Other	270 (6.6)	9 (7.0)	757 (8.7)	205 (9.2)	176 (7.3)	180 (7.5)	
BMI, mean (SD)	27.9 (5.7)	28.1 (4.7)	28.7 (6.2)	30.0 (5.9)	29.5 (6.30)	29.5 (5.49)	0.712
Education, no. (weighted %)							0.544^b^
<9 years	1,827 (12.9)	66 (12.7)	1,346 (6.6)	363 (7.6)	440 (18.3)	422 (17.6)	
>15 years	945 (23.2)	47 (24.5)	1,728 (28.1)	438 (26.7)	433 (18.0)	460 (19.2)	
9-15 years	3,875 (63.8)	181 (62.8)	5,282 (65.3)	1,388 (65.7)	1,528 (63.6)	1,519 (63.3)	
Unknown	0 (0.0)	0 (0.0)	7 (0.0)	1 (0.0)	0 (0.0)	0 (0.0)	
Hypertension, no. (weighted %)	2,682 (33.5)	177 (55.5)	3,417 (34.8)	1,427 (61.5)	1,136 (47.3)	1,544 (64.3)	<0.001
Diabetes, no. (weighted %)	959 (9.1)	62 (16.6)	1,016 (7.7)	616 (22.2)	395 (16.5)	649 (27.0)	<0.001
Currently smoking, no. (weighted %)	1,672 (24.4)	41 (11.5)	1,621 (19.5)	311 (15.3)	432 (18.0)	347 (14.5)	0.001
10-year ASCVD risk, mean (SD)	11.9 (12.1)	17.2 (13.0)	9.9 (11.2)	14.9 (12.5)	15.3 (13.0)	15.0 (12.5)	0.538
10-year ASCVD risk group, no. (weighted %)							<0.001
Under 5%	2,530 (48.2)	47 (26.0)	3,921 (58.6)	510 (32.4)	635 (26.4)	551 (22.9)	
5%-7.4%	731 (11.6)	32 (13.4)	956 (11.6)	261 (14.3)	223 (9.3)	287 (12.0)	
7.5%-20%	2,013 (26.9)	115 (32.6)	2,171 (20.6)	813 (34.3)	816 (34.0)	892 (37.2)	
20% and over	1,373 (14.4)	100 (28.0)	1,315 (9.2)	606 (19.1)	727 (30.3)	671 (27.9)	
Use of lipid-lowering therapy (weighted %)	4.5		19.7		NA		

All-cause mortality

**Figure 3:**
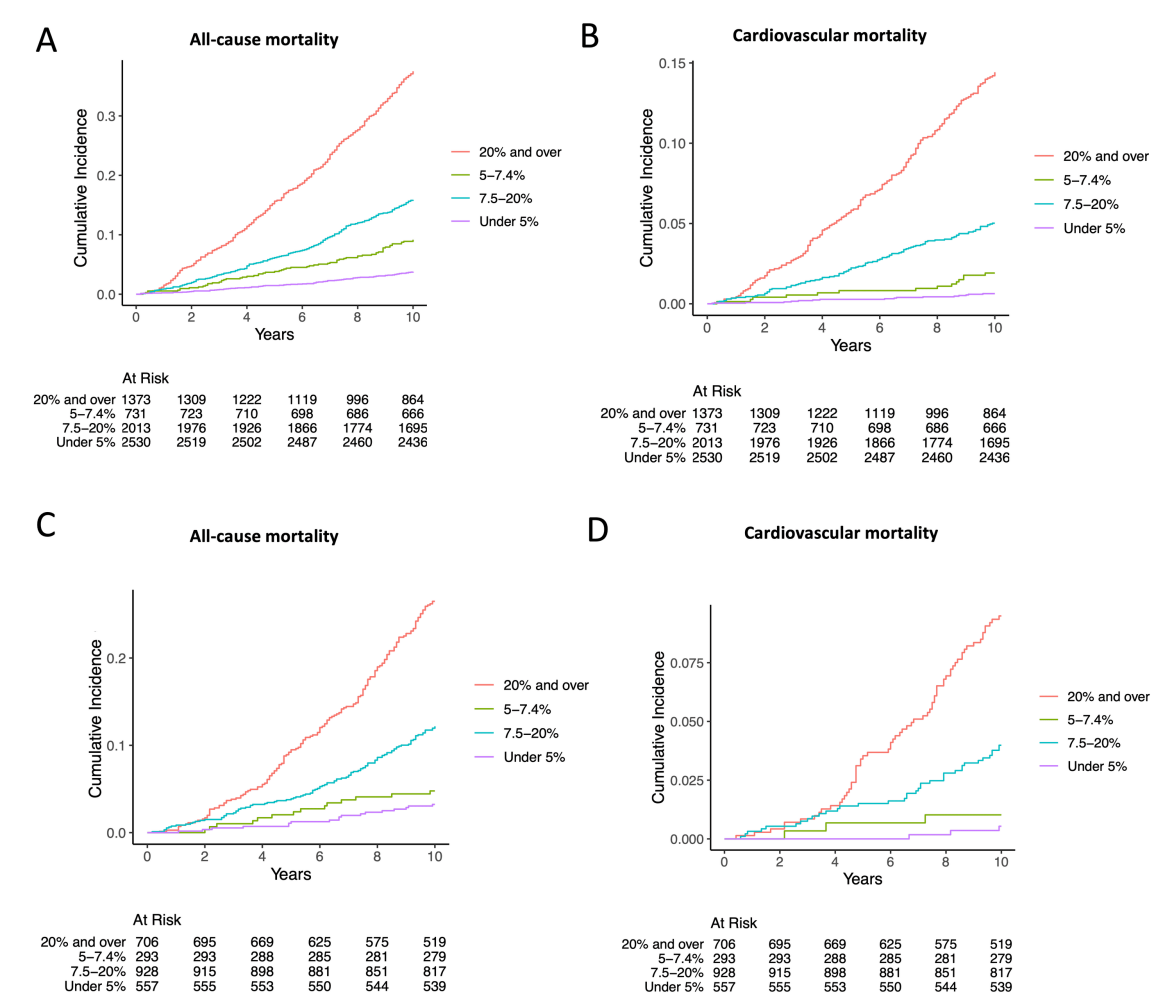
Cumulative incidence of each 10-year ASCVD risk group: (A) all-cause mortality in individuals who were not receiving lipid-lowering therapy; (B) cardiovascular mortality in individuals who were not receiving lipid-lowering therapy; (C) all-cause mortality in individuals who were receiving lipid-lowering therapy; (D) cardiovascular mortality in individuals who were receiving lipid-lowering therapy ASCVD: atherosclerotic cardiovascular disease

**Figure 4:**
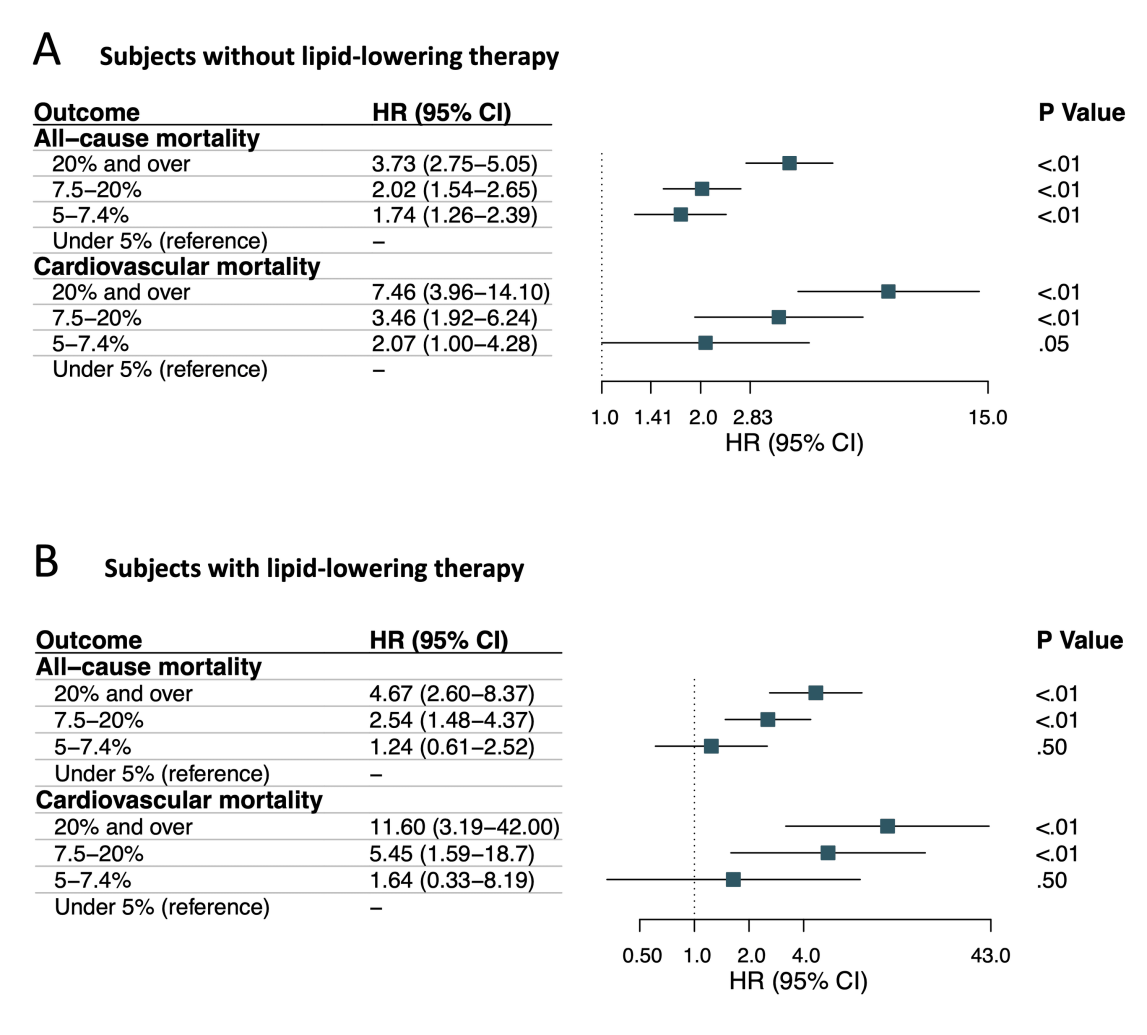
Hazard ratios for each 10-year ASCVD risk group: (A) without or (B) with lipid-lowering therapy using ASCVD risk < 5% group as the reference group ASCVD: atherosclerotic cardiovascular disease; HR: hazard ratio; CI: confidence interval

The incidence rate of all-cause mortality increased with the increasing ASCVD risk category (Figures 3A, 3C). Cox proportional hazards analyses, using 10-year ASCVD risk < 5% as the reference, revealed significant associations with all-cause mortality for ASCVD risk ≥ 5% to 7.4% (adjusted HR, 1.74; 95% CI, 1.26-2.39; p < 0.01), ≥7.5% to 19.9% (adjusted HR, 2.02; 95% CI, 1.54-2.65; p < 0.01), and ≥20% (adjusted HR, 3.73; 95% CI, 2.75-5.05; p < 0.01) in the control group (Figures 3A, 4A). Similarly, individuals with lipid-lowering therapy had an increased ASCVD risk ≥ 5% to 7.4%, ≥7.5% to 19.9%, and ≥20% with HR of 1.24 (95% CI, 0.61-2.52; p = 0.50), 2.54 (95% CI, 1.48-4.37; p < 0.01), and 4.67 (95% CI, 2.60-8.37; p < 0.01), respectively (Figures 3C, 4B). 

**Figure 5:**
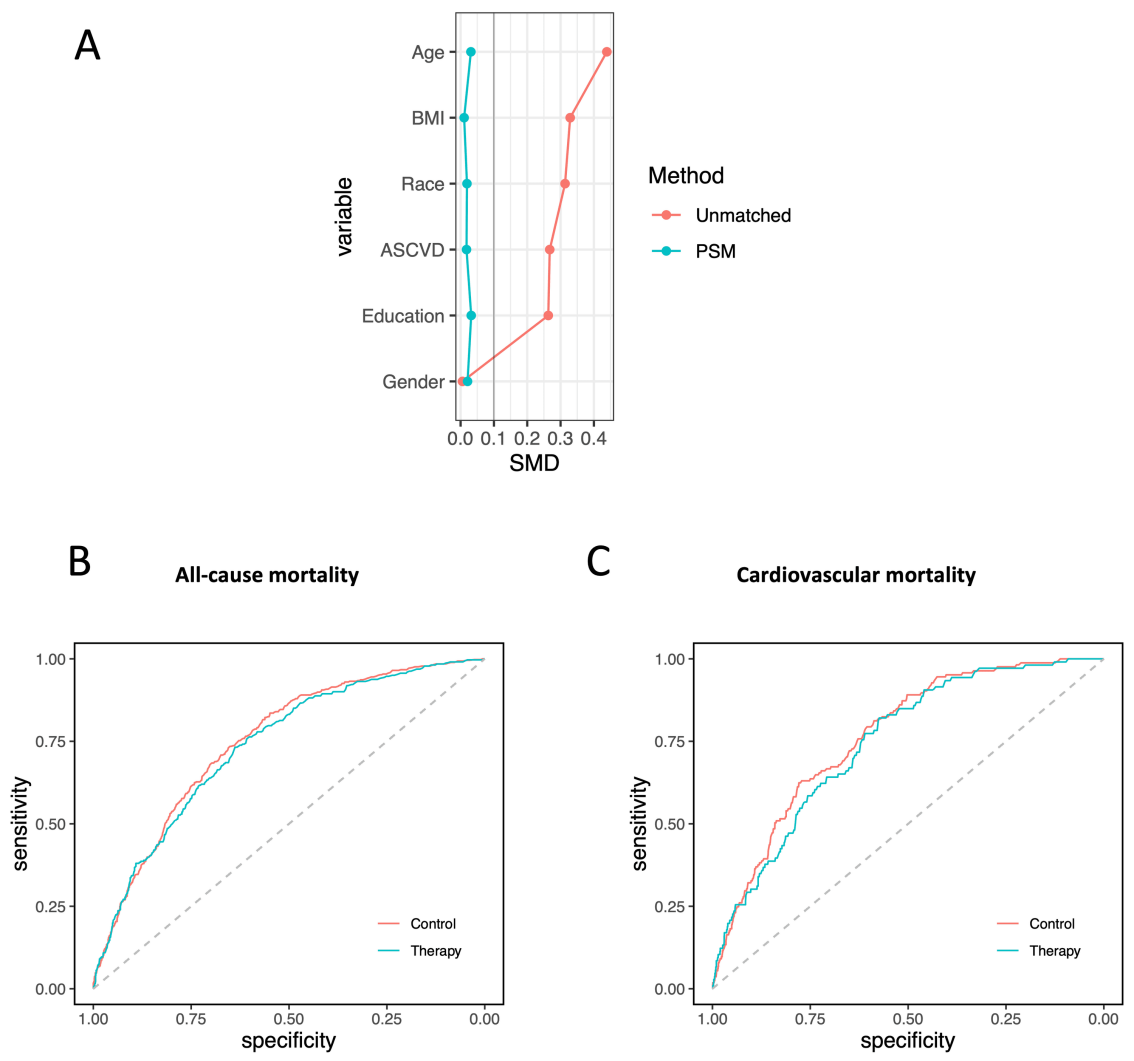
Receiver operating characteristic curve analysis of 10-year ASCVD risk to predict mortality in the matched cohort: (A) changes in standardized mean difference (SMD) before and after matching; (B) predicting all-cause mortality in untreated control and lipid-lowering therapy groups (AUC (95% CI): 0.74 (0.71-0.77) versus 0.75 (0.73-0.77); p = 0.50); (C) predicting cardiovascular mortality in untreated control and lipid-lowering therapy groups (AUC (95% CI): 0.77 (0.73-0.80) versus 0.75 (0.70-0.79); p = 0.47) ASCVD: atherosclerotic cardiovascular disease; AUC: area under the curve; PSM: propensity score matching; BMI: body mass index

**Figure 6:**
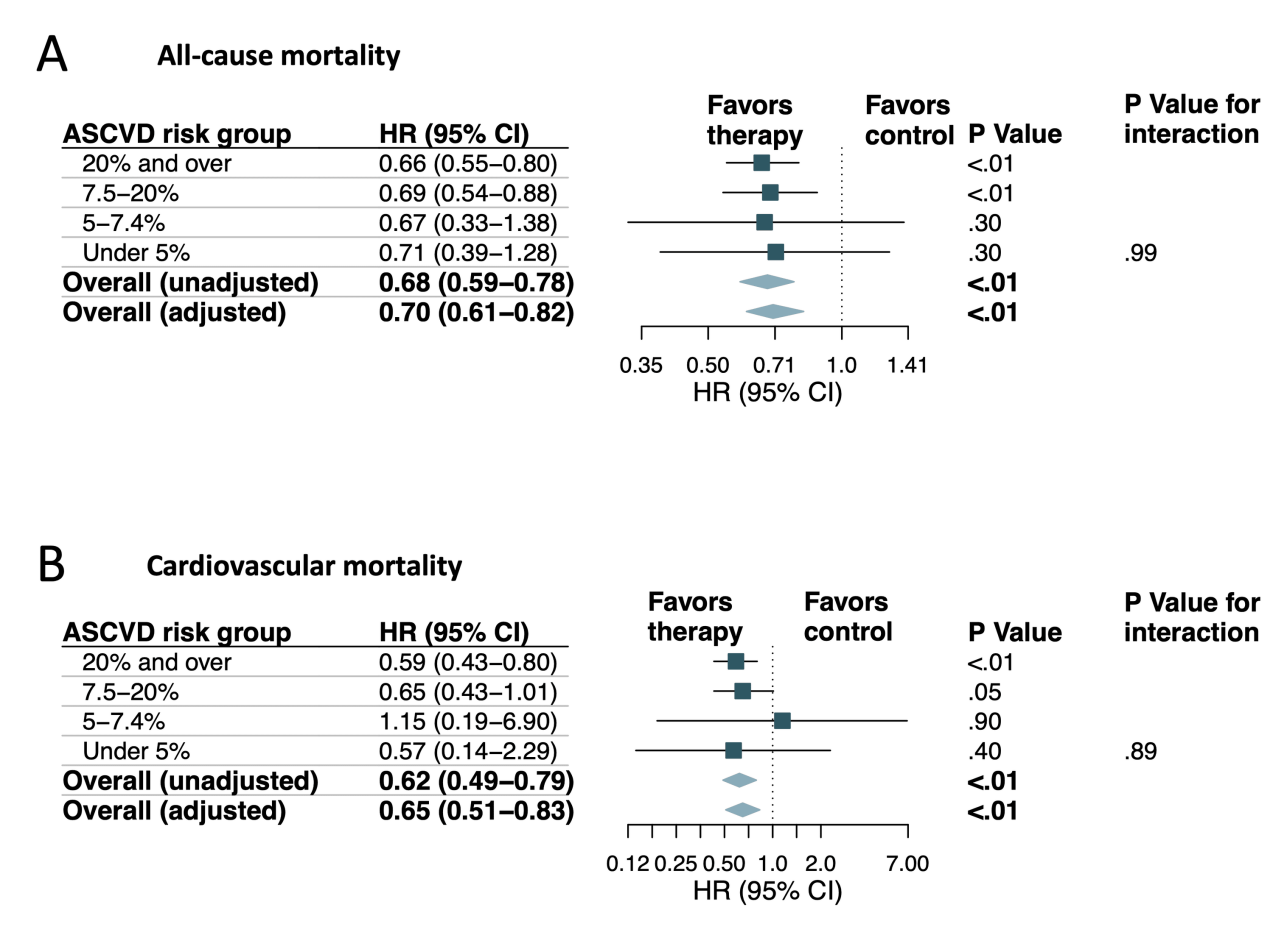
Analysis of mortality outcomes in the matched cohort: (A) hazard ratios and 95% confidence intervals for all-cause mortality are presented for the total cohort and subgroups categorized by increasing 10-year ASCVD risk; (B) hazard ratios and 95% confidence intervals for cardiovascular mortality are displayed for the total cohort and subgroups categorized by increasing 10-year ASCVD risk ASCVD: atherosclerotic cardiovascular disease; HR: hazard ratio; CI: confidence interval

**Figure 7:**
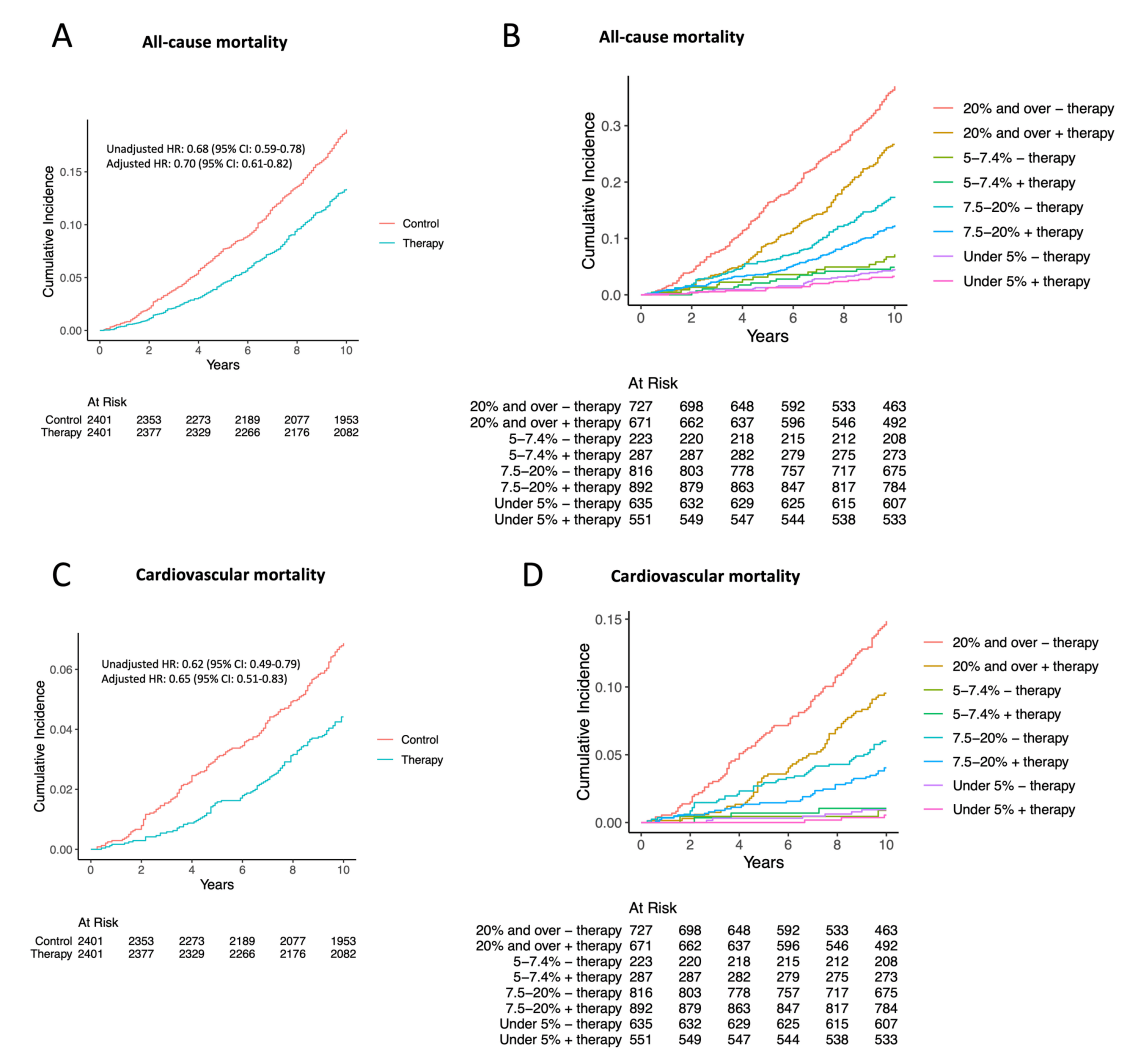
Cumulative event rate of primary and secondary outcomes in the matched cohort: (A) all-cause mortality in individuals who were receiving lipid-lowering therapy, compared to those who were not; (B) all-cause mortality in subgroups with increasing categories of 10-year ASCVD risk; (C) cardiovascular mortality in individuals who were receiving lipid-lowering therapy, compared to those who were not ASCVD: atherosclerotic cardiovascular disease; HR: hazard ratio; CI: confidence interval

In the propensity score-matched cohort, co-variables, including age, BMI, gender, race, education, and 10-year ASCVD risk, were well balanced (Figure 5A). The AUCs for 10-year ASCVD risk by PCE in the control and lipid-lowering therapy groups for predicting all-cause mortality were similar (0.74; 95% CI, 0.71-0.77 versus 0.75; 95% CI, 0.73-0.77; p = 0.50) (Figure 5B). These findings suggest that the predictive ability of the PCE for all-cause mortality is consistent across both groups, indicating that lipid-lowering therapy does not significantly alter the effectiveness of ASCVD risk assessment in predicting mortality. Lipid-lowering therapy was significantly associated with decreased all-cause mortality compared to control in the unadjusted analysis (HR, 0.68; 95% CI, 0.59-0.78; p < 0.01) (Figures 6A, 7A). The risk reduction remained statistically significant after multivariable adjustment (adjusted HR, 0.70; 95% CI 0.61-0.82; p < 0.01) (Figure 6A). In the subgroup analysis, individuals with a 10-year risk of ASCVD of 20% and over and ≥7.5% to 19.9% were associated with a significantly lower risk of all-cause mortality with HRs of 0.66 (95% CI, 0.55-0.80; p < 0.01) and 0.69 (95% CI, 0.54-0.88; p < 0.01) (Figures 6A, 7B). There was no significant interaction with 10-year ASCVD risk seen for lipid-lowering therapy status (p = 0.99 for interaction) (Figure 6A).

**Figure 8:**
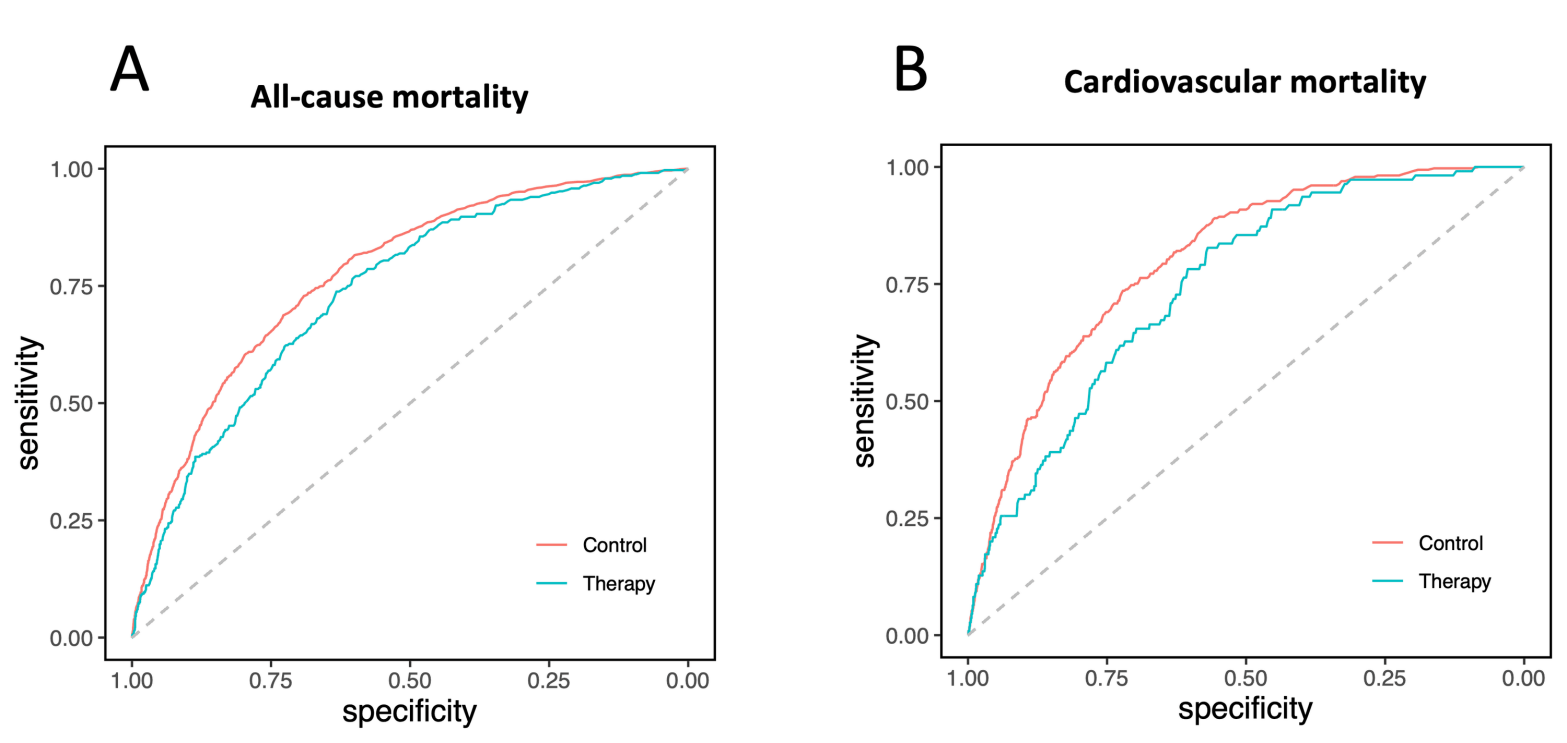
Receiver operating characteristic curve analysis of 10-year ASCVD risk to predict mortality in the unmatched cohort: (A) predicting all-cause mortality in untreated control and lipid-lowering therapy groups (AUC (95% CI): 0.78 (0.76-0.79) versus 0.74 (0.71-0.76); p = 0.02); (B) predicting cardiovascular mortality in untreated control and lipid-lowering therapy groups (AUC (95% CI): 0.80 (0.78-0.82) versus 0.75 (0.70-0.79); p = 0.02) ASCVD: atherosclerotic cardiovascular disease; AUC: area under the curve; CI: confidence interval

**Figure 9:**
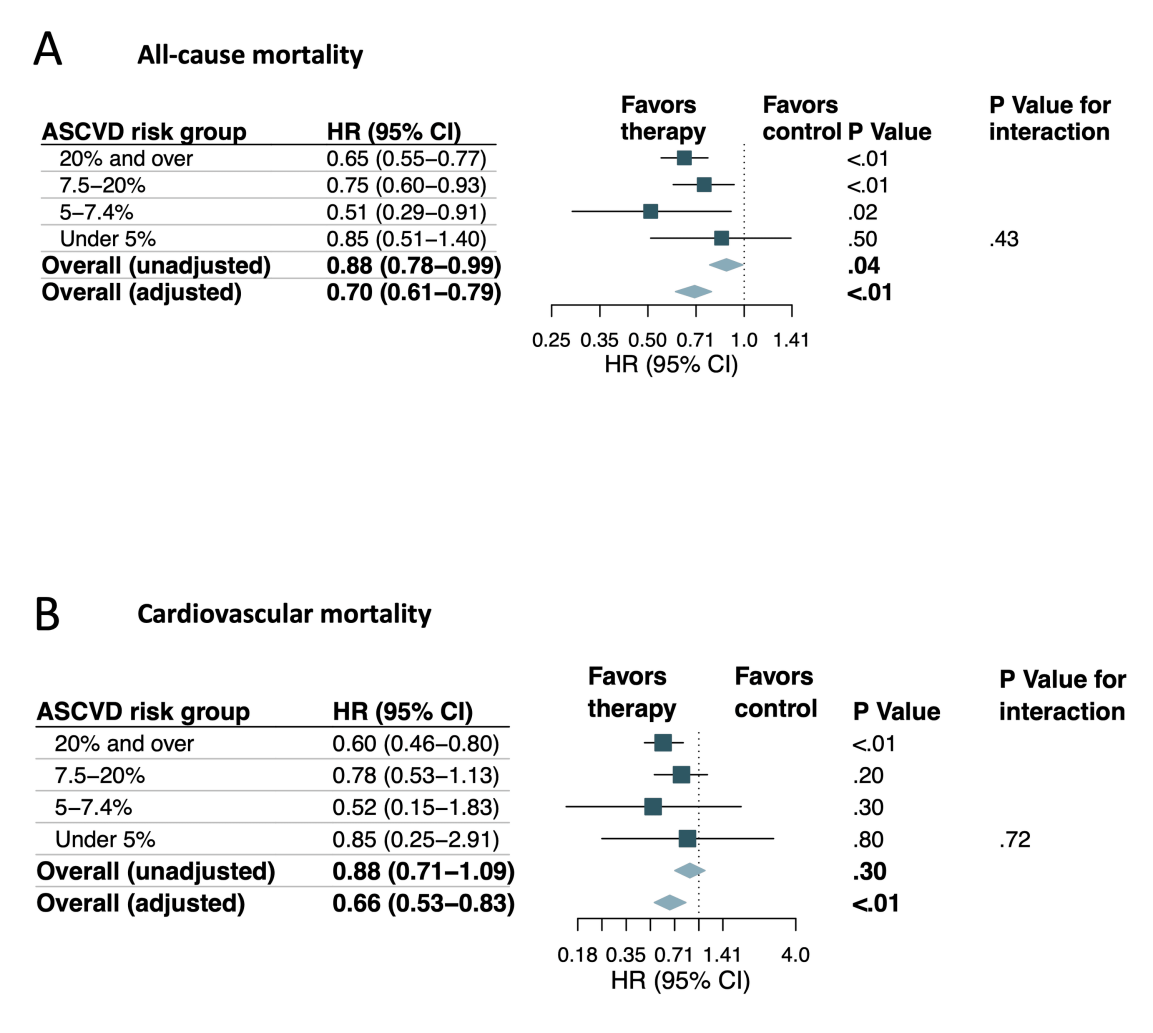
Analysis of mortality outcomes in the unmatched cohort: (A) hazard ratios and 95% confidence intervals are shown for all-cause mortality in the total cohort and subgroups with increasing categories of 10-year ASCVD risk; (B) hazard ratios and 95% confidence intervals are shown for cardiovascular mortality in the total cohort and subgroups with increasing categories of 10-year ASCVD risk ASCVD: atherosclerotic cardiovascular disease; HR: hazard ratio; CI: confidence interval

**Figure 10:**
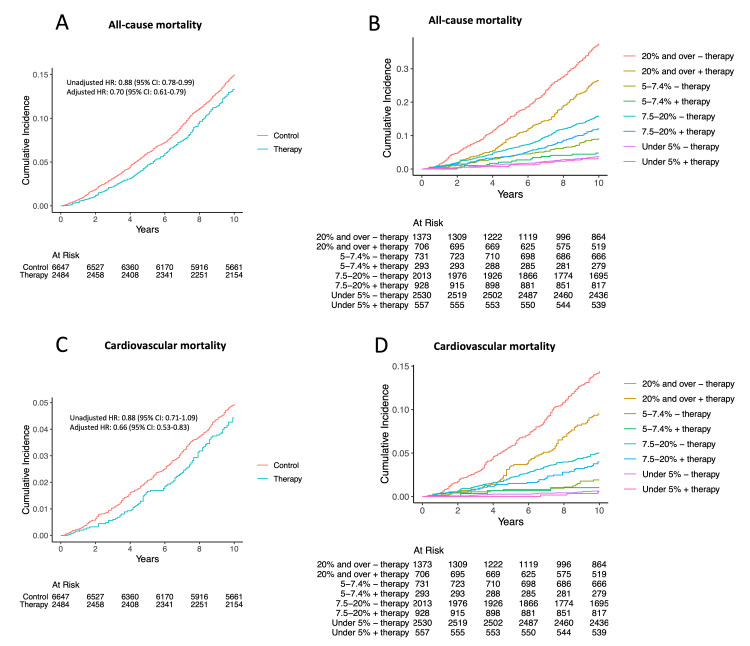
Cumulative event rate of primary and secondary outcomes in the unmatched cohort: (A) all-cause mortality in individuals who were receiving lipid-lowering therapy, compared to those who were not; (B) all-cause mortality in subgroups with increasing categories of 10-year ASCVD risk; (C) cardiovascular mortality in individuals who were receiving lipid-lowering therapy, compared to those who were not; (D) cardiovascular mortality in subgroups with increasing categories of 10-year ASCVD risk ASCVD: atherosclerotic cardiovascular disease; HR: hazard ratio; CI: confidence interval

In the unmatched cohort, the AUC for 10-year ASCVD risk by PCE in control for predicting all-cause mortality was slightly higher than that for lipid-lowering therapy groups (0.78; 95% CI, 0.76-0.79 versus 0.74; 95% CI, 0.71-0.76; p = 0.02) (Figure 8A). Additionally, lipid-lowering therapy was found to be significantly associated with reduced all-cause mortality in the unadjusted analysis when compared to the control group (HR, 0.88; 95% CI 0.78-0.99; p = 0.04) (Figures 9A, 10A). The risk reduction remained statistically significant after multivariable adjustment (adjusted HR, 0.70; 95% CI 0.61-0.79; p < 0.01) (Figure 9A). Furthermore, in the subgroup analysis, individuals with a 10-year risk of ASCVD of ≥20%, ≥7.5% to 19.9%, and ≥5% to 7.4% were associated with significantly lower all-cause mortality with HRs of 0.65 (95% CI, 0.55-0.77; p < 0.01), 0.75 (95% CI, 0.60-0.93; p < 0.01), and 0.51 (95% CI, 0.29-0.91; p = 0.02), respectively (Figures 9A, 10B). Notably, no significant interaction was observed with 10-year ASCVD risk for lipid-lowering therapy status (p = 0.43 for interaction) (Figure 9A).

Cardiovascular mortality

The incidence rate of cardiovascular mortality rose with increasing ASCVD risk category (Figures 3B, 3D). In Cox proportional hazards analyses, using 10-year ASCVD risk < 5% as the reference, there were significant associations with cardiovascular mortality for ASCVD risk ≥ 5% to 7.4% (adjusted HR, 2.07; 95% CI, 1.00-4.28; p = 0.05), ≥7.5% to 19.9% (adjusted HR, 3.46; 95% CI, 1.92-6.24; p < 0.01), and ≥20% (adjusted HR, 7.46; 95% CI, 3.96-14.10; p < 0.01) in the control group (Figures 3B, 4A). Similarly, increased risk of cardiovascular mortality was observed in individuals with lipid-lowering therapy for the ASCVD risk ≥ 5% to 7.4%, ≥7.5% to 19.9%, and ≥20% with HRs of 1.64 (95% CI, 0.33-8.19; p = 0.5), 5.45 (95% CI, 1.59-18.7; p < 0.01), and 11.6 (95% CI, 3.19-42.00; p < 0.01), respectively (Figures 3D, 4B).

In the propensity score-matched cohort, the AUCs for 10-year ASCVD risk by PCE in the control and lipid-lowering therapy groups for predicting cardiovascular mortality were similar (0.77; 95% CI, 0.73-0.80 versus 0.75; 95% CI, 0.70-0.79; p = 0.47) (Figure 5C). Compared to the control, lipid-lowering therapy was significantly associated with decreased cardiovascular mortality in the unadjusted analysis (HR, 0.62; 95% CI 0.49-0.79; p < 0.01) (Figures 6B, 7C). Risk reduction remained statistically significant after multivariable adjustment (adjusted HR, 0.65; 95% CI 0.51-0.83; p < 0.01) (Figure 6B). In the subgroup analysis, individuals with a 10-year risk of ASCVD of 20% and over were associated with a significantly lower risk for cardiovascular mortality with a HR of 0.59 (95% CI, 0.43-0.80; p < 0.01) (Figures 6B, 7D). The test for interaction between lipid-lowering therapy status and 10-year ASCVD risk was not significant (p = 0.89 for interaction) (Figure 6B).

In the unmatched cohort, the AUC for 10-year ASCVD risk in the control group for predicting cardiovascular mortality was slightly higher compared to that for lipid-lowering therapy groups (0.80; 95% CI, 0.78-0.82 versus 0.75; 95% CI, 0.70-0.79; p = 0.02) (Figure 8B). In the unadjusted analysis, lipid-lowering therapy was not significantly associated with decreased cardiovascular mortality compared to the control group (HR, 0.88; 95% CI 0.71-1.09; p = 0.30) (Figures 9B, 10C). However, after multivariable adjustment, the risk reduction was statistically significant (adjusted HR, 0.66; 95% CI 0.53-0.83; p < 0.01) (Figure 9B). In the subgroup analysis, individuals with a 10-year risk of ASCVD of ≥20% were associated with significantly lower cardiovascular mortality with a HR of 0.60 (95% CI, 0.46-0.80; p < 0.01) (Figures 9B, 10D). There was no significant interaction with 10-year ASCVD risk observed for lipid-lowering therapy status (p = 0.72 for interaction) (Figure 9B).

## Discussion

This study based on NHANES participants from the period 1988-1994 and 1999-2008 found that the ability of 10-year ASCVD risk using the PCE to predict all-cause and cardiovascular mortality was comparable for individuals who were receiving lipid-lowering therapy and those who were not, after adjusting for the propensity to receive lipid-lowering therapy. Furthermore, there was an association between lipid-lowering therapy and the reduction of all-cause and cardiovascular mortality, with the greatest reductions for individuals with a 10-year ASCVD risk of ≥7.5%.

In clinical practice, the 10-year risk of ASCVD calculated using the PCE has been used to identify individuals who are at high risk of ASCVD, but its prognostic value for lipid-lowering therapy has not been explored. It is crucial to use the same metric to assess the effectiveness of lipid-lowering therapy on all-cause and cardiovascular mortality for individuals before and after therapy. To determine the association of 10-year ASCVD risk and baseline mortality with minimal confounding from lipid-lowering therapy, eligible individuals without lipid-lowering therapy from NHANES III were included in this study, as a significant increase in lipid-lowering therapy was noted in the USA from the period 1984-1994 (NHANES III) to the early 2000s, which is in accordance with prior findings that the use of lipid-lowering therapy increased significantly after the early 2000s [21]. For comparison, individuals with lipid-lowering therapy from the early 2000s were included. However, the limitations of this approach are discussed below.

PCEs have previously shown moderate to good discrimination for ASCVD mortality, with reported AUCs of 0.716 (95% CI, 0.663-0.770) for non-Hispanic White, 0.794 (95% CI, 0.734-0.854) for non-Hispanic Black, and 0.733 (95% CI, 0.654-0.811) for Mexican American populations, based on data from NHANES 1988-1994 in individuals not receiving lipid-lowering therapy [22]. Consistent with these findings, our analysis demonstrated that the AUC for PCE in predicting cardiovascular mortality at 10 years for individuals not on lipid-lowering therapy was 0.77 (95% CI, 0.73-0.80) in the propensity score-matched cohort and 0.78 (95% CI, 0.76-0.79) in the unmatched cohort. Additionally, a previous study has shown that the ASCVD risk score estimated by PCE is associated with both all-cause and cardiovascular-specific mortality and that individuals with a 10-year ASCVD risk of ≥7.5% have been shown to have a threefold increased risk of cardiovascular mortality compared to those with a risk of <7.5% [23]. Similarly, in our study, we found a significant 2.1-fold, 3.5-fold, and 7.5-fold increased risk of cardiovascular mortality for individuals with ASCVD risks of 5% to 7.4%, 7.5% to 19.9%, and ≥20%, respectively, compared to those with a risk of <5%. Statins have been widely used as the primary lipid-lowering drugs. Several clinical trials and meta-analysis have demonstrated their beneficial effects on all-cause mortality and cardiovascular mortality in individuals with or without cardiovascular disease [11,13-15,17,24-26]. Consistent with this, we found that lipid-lowering therapy was significantly associated with a 30% reduction in all-cause mortality and a 35% reduction in cardiovascular mortality in matched individuals with comparable 10-year risk of ASCVD. In the subgroup analysis, the greatest reductions were observed in individuals with a 10-year ASCVD risk of ≥7.5%. Importantly, the all-mortality benefit remained significant in the unmatched cohort analysis, providing further evidence of the effectiveness of lipid-lowering therapy in reducing all-cause mortality. These results provide a basis for assessing mortality risk reduction using 10-year ASCVD risk by PCE as a key tool in individuals before and after lipid-lowering treatment, in addition to LDL-C levels. Future studies are needed to further validate our findings with other datasets.

It's crucial to recognize that the PCE was derived to gauge the 10-year risk of clinical ASCVD. While there have been reports indicating an overestimation of ASCVD relative risk by these equations [27,28], recent studies have demonstrated their robust performance within real-world community cohorts, irrespective of age, blood pressure, blood cholesterol levels, or the initiation of statin therapy during follow-up [29]. These findings have further strengthened the utilization of PCE in assessing ASCVD risk. Our data corroborate these findings, demonstrating that the PCE consistently predicts both all-cause and cardiovascular mortality regardless of lipid-lowering therapy status. This strengthens the case for employing the 10-year ASCVD risk estimated by PCE to monitor the relative reduction in mortality risk associated with lipid-lowering therapy.

The present study has several limitations. First, the lack of subsequent data on the initiation and discontinuation of lipid-lowering therapy after the initial interview in NHANES could have confounded the observed benefits. Second, a considerable number of participants did not provide responses regarding their lipid-lowering therapy status in NHANES, which could introduce bias when assuming nontherapy status for analyses. However, similar conclusions were obtained when analyses were restricted to individuals who explicitly reported receiving lipid-lowering therapy or not (data not shown). Third, the study did not document specific medications for lipid-lowering therapy, which may have included classes of medication beyond statins. Fourth, despite the use of propensity score matching and multiple variable adjustments to balance and control for potential confounding factors, residual confounders are likely to exist and could not be fully accounted for in this retrospective cohort study. Lastly, the sample size of the subgroup with borderline risk (5%-7.4%) may have been too small to detect a statistically significant difference, indicating the need for further investigation on whether lipid-lowering therapy provides greater benefits to individuals with borderline ASCVD risk after treatment compared to those who do not receive such treatment.

## Conclusions

The results of this study using NHANES data with a follow-up period of at least 10 years show that the 10-year risk of ASCVD calculated using the PCE has a comparable prognostic value for all-cause and cardiovascular mortality, irrespective of lipid-lowering therapy status. Additionally, our findings demonstrate that lipid-lowering therapy is associated with a reduction in both all-cause and cardiovascular mortality in individuals receiving treatment, particularly among those with a 10-year ASCVD risk of 7.5% or higher, compared to untreated individuals with a similar or higher ASCVD risk. These results highlight the great potential of using the 10-year risk of ASCVD using the PCE for assessing mortality risk reduction in individuals before and after treatment for the prevention of ASCVD.
